# Brief gatekeeper training for suicide prevention in an ethnic minority population: a controlled intervention

**DOI:** 10.1186/s12888-016-0924-4

**Published:** 2016-07-07

**Authors:** Alan R. Teo, Sarah B. Andrea, Rae Sakakibara, Satoko Motohara, Monica M. Matthieu, Michael D. Fetters

**Affiliations:** VA Portland Health Care System, Health Service Research & Development (HSR&D) Center to Improve Veteran Involvement in Care (CIVIC), 3710 SW US Veterans Hospital Rd (R&D 66), Portland, OR 97239-2964 USA; Department of Psychiatry, Oregon Health & Science University, 3181 SW Sam Jackson Park Rd (Multnomah Pavilion, Room 2316), Portland, OR 97239-3098 USA; Oregon Health & Science University and Portland State University, School of Public Health, 506 SW Mill St., Suite 450 (OMPH-SCH), Portland, OR 97201 USA; Department of Family Medicine, University of Michigan, 1018 Fuller Street, Ann Arbor, MI 48104-1213 USA; Department of Veterans Affairs, Central Arkansas VA Health Care System, Mental Health Services and VA Mental Health Quality Enhancement Research Initiative, 2200 Fort Roots Dr., Bldg. 58, North Little Rock, 72114, Little Rock, AR 72205 USA; Saint Louis University, College for Public Health and Social Justice, School of Social Work, Tegeler Hall, 3550 Lindell Blvd, Suite 300, St. Louis, MO 63106 USA

**Keywords:** Gatekeeper training, Suicide prevention, Identification and referral, Intervention, Japanese, Asian, Community, Mixed methods

## Abstract

**Background:**

Suicide is a critical public health problem around the globe. Asian populations are characterized by elevated suicide rates and a tendency to seek social support from family and friends over mental health professionals. Gatekeeper training programs have been developed to train frontline individuals in behaviors that assist at-risk individuals in obtaining mental health treatment. The purpose of this study is to assess the efficacy of a brief, multi-component gatekeeper intervention in promoting suicide prevention in a high-risk Asian community in the United States.

**Methods:**

We adapted an evidence-based gatekeeper training into a two-hour, multi-modal and interactive event for Japanese-Americans and related stakeholders. Then we evaluated the intervention compared to an attention control using mixed methods.

**Results:**

A sample of 106 community members participated in the study. Intervention participants (*n* = 85) showed significant increases in all three types of intended gatekeeper behavior, all four measures of self-efficacy, and both measures of social norms relevant to suicide prevention, while the control group (*n* = 48) showed no significant improvements. Additional results showed significantly higher satisfaction and no adverse experiences associated with the gatekeeper training. The separate collection of qualitative data, and integration with the quantitative survey constructs confirmed and expanded understanding about the benefits of the intervention.

**Conclusions:**

A brief, multi-modal gatekeeper training is efficacious in promoting positive gatekeeper behaviors and self-efficacy for suicide prevention in an at-risk ethnic minority population of Japanese Americans.

## Background

Suicide is a critical public health problem around the globe [1]. In the United States (U.S.), it is a leading source of mortality [2], and the rate of suicide in other countries, notably Japan, has also increased in the last two decades. Data from 2011 show Japan’s rate of 18.2 suicide deaths per 100,000 people is nearly 50 % higher than the U.S. rate of 12.3 in the same year [3, 4]. As a multicultural country, ethnic minority populations in the U.S. are of major importance, and Census data indicate the Asian population (referring to persons having origins in any of the original peoples of the Far East, Southeast Asia, or the Indian Subcontinent) is growing faster than all other racial groups. Japanese-Americans comprise 1.3 of the 17.3 million Asians counted in the 2010 U.S. Census [5].

Recently, experts in suicide prevention have emphasized a need to focus suicide prevention efforts on strengthening social bonds and community connectedness [2]. This approach is necessary given that an individual in crisis may not seek professional help when left to his or her own devices. Indeed, Asians (person having origins in any of the original peoples of the Far East, Southeast Asia, or the Indian Subcontinent) are the ethnic group in the U.S. least likely to have a health care visit preceding a suicide attempt [6]. When an at-risk person does seek professional mental health referral or treatment, family and friends frequently play a key role in convincing the person to do so [7]. In Japan too, individuals with suicidal ideation are likely to seek advice from close members of their social network [8]. However, even with the best of intentions, family, friends, or other members of the community in contact with an individual at risk for suicide are not naturally adept in providing assistance and referral [9].

Training concerned community members so they are equipped to provide direct, specific, and supportive assistance to an at-risk individual is a promising approach for which the Institute of Medicine has recommended expansion [10]. While community-based suicide prevention interventions have often focused on providing information and education in a lecture-style format [11], experiential educational components appear more effective (W. F. [12, 13]). For instance, a randomized controlled trial of gatekeeper training for school teachers and parents found that behavioral rehearsal with role play practice resulted in higher total gatekeeper skill scores immediately after training and at follow-up, compared with training as usual lacking the behavioral rehearsal [12]. A number of interventions—often collectively referred to as gatekeeper training programs—have been developed to train frontline individuals, or gatekeepers, in assisting an at-risk person to identify supportive resources. The programs include Mental Health First Aid, Applied Suicide Intervention Skills Training (ASIST), Signs of Suicide (SOS), and Question, Persuade, and Refer (QPR) [14–17].

QPR consists of a brief standardized role-play exercise and a 1 h didactic training with a short video that teaches three gatekeeper skills (ie, questioning, persuading, and referring). The QPR program has been reviewed by the Substance Abuse and Mental Health Services Administration’s (SAMHSA) National Registry of Evidence-based Programs and Practices, and empirical evaluations have shown improvements in suicide-related knowledge and self-efficacy [17–19]. Additionally, the interactive role play exercise has been empirically tested with gains in suicide-specific skills at post-training [20, 21].

Video, or film, may also be an effective educational tool because it engages the individual’s senses (eg, visual and auditory), more than print media [22], and it can help shape social norms and health behaviors [23]. A Japanese study found that community residents who watched a publicly-distributed educational video on suicide prevention endorsed more awareness of professional mental health referral resources and having a larger network of people available for help-seeking compared to non-watching residents [8].

While previous evaluations using QPR and role play have demonstrated gains in knowledge, self-efficacy and gatekeeper specific skill outcomes in before-and-after uncontrolled studies, few studies have utilized more rigorous study designs. Even less is known about the impact of cultural adaptations to high-risk ethnic minority populations. Therefore, the a priori primary aim of this controlled study was to determine if participation in a gatekeeper training intervention including components of QPR, video and active learning, and adapted to target Japanese-Americans and related stake holders population would increase intended gatekeeper behavior and self-efficacy related to suicide prevention. Our exploratory aims were to determine if participation would change social norms or include negative experiences for participants.

## Methods

### Participants

The intervention and control events were free and open to the public. This study was granted an exemption from ethics approval – by the Institutional Review Board of VA Portland Health Care System – since survey data were collected anonymously. The event targeted Japanese Americans, expatriates and related stakeholders, ie, non-Japanese descent individuals interested in Japanese culture (elsewhere abbreviated as Japanese Americans). Anyone who voluntarily came to the event was included in the study, and anyone who opted not to complete the survey was excluded. The events were entitled “Saving 10,000” and promoted using mailing lists, posters, flyers, and articles in the local press (both English and Japanese print media) by a university center dedicated to Japanese studies. The control was conducted during a single event in English, while the intervention was conducted as two events, once in English and held on a university campus, and once in Japanese and held in a community venue. The venue in English attracted roughly half Japanese and half non-Japanese participants, while the Japanese venue attracted essentially 100 % Japanese participants. The control was conducted as one event in English and held on a university campus.

### Multi-component intervention

To help develop our adaptation of the intervention we first conducted pilot testing. The pilot paralleled the current study in three critical ways: first, it targeted the same population (Japanese Americans) as the current study, second, it addressed potentially stigmatizing mental health issues, and, third, tested a three-component intervention (didactic lecture, film screening, and question-and-answer session). Results from a pre and post survey with responses from 115 participants indicated that 90 % intended to get more information about, 48 % to be vigilant for, and 20 % to talk with a health care professional about mental health concerns in themselves or someone they knew [24].

In the present study, we maintained a multi-component format for the intervention. Our intervention, though novel, drew from the literature on gatekeeper training, which can include any combination of multimedia or video presentation, didactic training, or interactive components including behavioral rehearsal [14, 19]. The film screening occurred first, followed immediately by an experiential educational session, and finally an interactive question-and-answer session. The film screened was entitled *Saving 10,000*, a 50-min bilingual (English and Japanese) documentary that provides a thoughtful exploration of the suicide epidemic in Japan [25]. The interactive training session was based on QPR [19]. Specifically, participants listened to a 15-min lecture by psychiatrist (A.T.) that described: 1) how to directly ask a person about having thoughts of suicide, 2) how to persuade a person to accept help, and 3) how to make a specific referral to professional assistance, including provision of local and national resources. Participants then received handouts on QPR gatekeeper skills, formed dyads, and practiced the skills in a 15-min role play using a scripted scenario and skill feedback form. Finally, for the question-and-answer session, a moderator collected written questions from audience members that were then directed to a panel of four bicultural and bilingual experts (a psychiatrist, psychologist, medical interpreter, and a family physician), which lasted approximately 30 min.

### Attention control

The control condition consisted of a lecture on the cultural context of suicide in Japan. Specifically, four university faculty members provided brief presentations examining suicide in Japan from the viewpoint of history, anthropology, contemporary medical practice, and literature. The lecture was followed by a moderated question-and-answer session. The control event was conducted contemporaneously with the intervention, received similar publicity and promotion, and was of similar duration (1.5 h) to the intervention event.

### Measures

A pre-post survey containing sections on background and demographic information, outcomes, and event feedback was administered to both groups. Outcome measures were largely based on similar questions used in prior studies of gatekeeper training and peer recognition and response to suicidal behavior [19, 26, 27].

The survey was developed in English and a Japanese version was administered at the Japanese community event. The Japanese survey adaptation process followed a stepwise process of creating two independent translations (S.M. and R.S.), comparing and reconciling versions (S.M. and R.S.), back-translating (R.S.), and finally comparing and reconciling significant differences (S.M., R.S., A.T., and M.F.).

#### Gatekeeper behavior

We used three individuals items that assessed how likely the participant would be to do the following suicide-specific skills taught in the QPR gatekeeper training for a close person at risk for suicide: “How likely would you be to: directly ask about suicidal thoughts; encourage to seek mental health treatment; and provide a specific phone number to get help.” Response choices were on a five-point scale from 1 (“very unlikely”) to 5 (“very likely”). Items were combined into a gatekeeper behavior scale that demonstrated adequate internal reliability (scale range: 1–5 points; α = 0.63).

#### Self-efficacy

We used four individual items that assessed how much the participant agreed with the following statements: “I am confident in helping someone with a mental health concern”; “It would be easy for me to directly ask someone close to me if he or she is thinking about suicide”; “If someone close to me is having a mental health problem, I can persuade him or her to get help”; “I know how to tell if someone close to me is at risk for suicide”. Response choices were on a five-point scale from 1 (“strongly disagree”) to 5 (“strongly agree”). Items were also combined into a self-efficacy scale that demonstrated adequate internal reliability (scale range: 1–5 points; α = 0.79).

#### Social norms

Based on prior research suggesting that social norms around mental health issues are more linked to the opinions and beliefs of close, or proximal, social contacts [28, 29], we constructed two items that determined agreement that most people close to them would: “seek help from a health care provider if they were thinking about suicide” (proximal descriptive social norm); and “approve of seeking help from a health care provider if they were thinking about suicide” (proximal injunctive social norm). Response choices were again on the same five-point scale as for self-efficacy. The items were also combined into a social norms scale that demonstrated adequate internal reliability (scale range: 1–5 points; α = 0.74).

#### Personal impact

On the post-event survey only, participants were asked three items inquiring if attending the event made them uncomfortable, depressed, or anxious, with response choices on the same five-point disagree-agree scale.

#### Qualitative data

Open-ended, written qualitative comments were solicited only for the intervention condition using three prompts: 1) “What part(s) of the film really stand out in your mind? 2) “What message in the film did you take away from watching it?; and 3) “If you have any other comments you would like to share (including ways to improve an event like this in the future), feel free to include them.” Ten pages of single-spaced comments were collected.

### Data analysis

The data analysis for this convergent mixed methods study involved two types of analysis. For quantitative analyses, we first compared the baseline characteristics of the control and intervention groups using means with standard deviations for continuous variables and frequencies with percentages for categorical variables. Similarly, we compared mean item scores between control and intervention groups using Fischer’s exact test for categorical variables and t-tests for continuous variables. Next, we compared the within-group pre and post individual item and scale means, using t-tests. For these comparisons, we also conducted a sensitivity analysis. Because the larger size of the intervention group biases the study to find significant effects in this group, we took a randomly generated subsample of intervention participants equal in number to the control group (*n* = 39 each).

To assess the impact of the intervention on the change in scale scores from pre- to post-event for gatekeeper behavior, self-efficacy, and social norms, we constructed unadjusted and adjusted regression models. In unadjusted models, study group and pre-event scale score were independent variables. To build adjusted models, we used purposeful forward stepwise selection. Specifically, all baseline characteristics were examined as potential confounders by first adding each potential confounder one at a time to the unadjusted model and rating its individual impact on percent change in the intervention coefficient. Composite models were then created by adding in each potential confounder in order of impact on intervention coefficient (largest first) and retaining them in the composite model if the addition changed the intervention coefficient by 10 % or more. Once a variable was entered in to the model, it was not removed. Regression diagnostic procedures were performed to establish linearity, normality, and equal variance of the final models and to assess the models for multicollinearity and influential data points. Missing responses were less than 10 % for all survey items included in these analyses. All statistical tests were two-tailed. Significance was considered at *P* < .05. Statistical analyses were performed by using Stata 13.1 (StataCorp, College Station, TX).

Analysis of the qualitative comments was conducted as follows. First, Japanese language text was translated by a bilingual research assistant, then reviewed for accuracy and revised by a second bilingual research assistant. Next, all text along with participants’ demographic information was imported into MAXQDA qualitative analysis software. Then, two research assistants independently coded text from five participants using a preliminary coding scheme designed for integration by using codes equivalent to key constructs in the structured survey items. Differences in interpretation were few, and managed by negotiating consensus between the two raters to calibrate coding procedures. They revised the coding scheme iteratively to include emerging concepts as codes as well. This double-coding and revision process was repeated for another set of 10 participants, with additional attention to identification of de novo themes. To further identify patterns, we also created matrices of text organized by the question being asked and participants’ race. When it was clear coding was consistent and calibrated, all remaining text was coded by a single research assistant (R.S.). The final version of the codebook included eight categories: gatekeeper behaviors, self-efficacy, social norms, communal efforts, awareness and knowledge, satisfaction, personal impacts, and suggestions.

Mixed methods data integration was achieved through linking of qualitative themes with the quantitative constructs from the survey through use of joint displays. Integration is a hallmark of mixed methods investigation quality [30]. Joint displays juxtapose representative quantitative and qualitative in a single table or figure [31]. This allows meta-inferences, namely, interpretations as to whether both types of data confirm, expand, or are discordant with each other [32].

## Results

Eighty-five people attended the intervention events (56 at the English event and 29 at the Japanese event) and 48 the control event. Of these, 108 completed surveys (response rate = 81.2 %) and two were ineligible for analysis (one each for attending a prior event and for providing incomplete responses), leaving a total sample of 106 participants. Table 1 shows that participants were generally middle-aged, highly educated, Japanese American, and members of the lay community. There were no statistically significant differences in characteristics of participants in the intervention and control groups except that control participants had on average one more year of education than intervention participants (*p* = 0.03).

**Table 1:** Baseline characteristics of participants by study group

	Control (*n* = 39)	Intervention (*n* = 67)	*P*-value*
	mean ± SD or n(%)		
Age	45.13 ± 17.84	45.00 ± 15.57	0.97
Female Gender	26 (68.42)	43 (66.15)	1.00
Race			
*White non-Hispanic*	8 (21.05)	18 (29.03)	0.20
*Japanese non-Hispanic*	18 (47.37)	34 (54.84)	
*Mixed or other*	12 (31.58)	10 (16.13)	
Education, in years	18.16 ± 2.43	17.05 ± 2.57	0.03
Health care provider	6 (15.79)	13 (20.00)	0.79
Number of close relations perceived as ever having been at risk for suicide			
*None*	9 (24.32)	23 (37.10)	0.60
*One*	11 (29.73)	14 (22.58)	
*Two or more*	12 (32.43)	17 (27.42)	
*Unsure*	5 (13.51)	8 (12.90)	
Seen or talked to a mental health provider in the last year	8 (21.62)	15 (23.81)	1.00

*T-tests for continuous variables, Fisher’s exact for categorical variables

Eight-six percent of the intervention participants strongly agreed that the event was excellent, whereas only 49 % of the control group strongly agreed with the same statement (*p* < 0.001). This finding was confirmed by qualitative comments of 18 participants (27 %) who volunteered their satisfaction with the event (eg, “Thank you. I enjoyed [the event] and felt time [was] well spent.” [E2_035]). The qualitative findings expanded understanding about their perspectives of the intervention. For example, several participants suggested allocating more time for the role play. On average, participants denied that the event made them uncomfortable, depressed, or anxious, just one qualitative comment alluded to the “intensity” of the topic, and no significant differences in negative personal impacts were found between intervention and control groups.

Table 2 summarizes and integrates the quantitative and qualitative results regarding gatekeeper behavior. Intervention participants showed significant improvement in their likelihood of using overall gatekeeper behavior and all three specific behaviors (questioning, persuading, and referring), while control participants showed no changes. Results of our sensitivity analysis were similar for overall and individual gatekeeper behaviors except for questioning, which became marginally significant (*p* = 0.10). Forty-three (64 %) participants also furnished qualitative comments that confirmed learning of helpful gatekeeper behaviors.

**Table 2:** Joint display linking quantitative results and qualitative comments related to intended gatekeeper behavior

Variable	Group	Pre score	Post score	*P*-value	Comments about intervention
Gatekeeper behavior (total)	Control	4.04 ± 0.78	4.09 ± 0.75	0.80	“Stop to help. Ask and show care and concern.” (E2_007) “Reach out and listen.” (J1_018) "Actions such as reaching out, being there for someone can help prevent even one person thinking about suicide from killing him/herself." (J1_002)
	Intervention	4.02 ± 0.90	4.57 ± 0.62	<0.001	
Directly ask about suicidal thoughts (Question)	Control	3.38 ± 1.48	3.59 ± 1.37	0.55	
	Intervention	3.66 ± 1.43	4.25 ± 1.11	0.01	
Encourage to seek mental health treatment (Persuade)	Control	4.49 ± 0.72	4.44 ± 0.75	0.79	
	Intervention	4.38 ± 0.98	4.74 ± 0.57	0.01	
Provide a specific phone number to get help (Refer)	Control	4.26 ± 0.91	4.23 ± 0.92	0.92	Mixed methods interpretation
	Intervention	4.01 ± 1.12	4.76 ± 0.61	<0.001	Qualitative comments confirm quantitative results.

Score range was on a five-point scale from 1 (“very unlikely”) to 5 (“very likely”)

Table 3 synthesizes quantitative and qualitative results regarding self-efficacy. Intervention participants showed significant improvement in overall and all four individual components of self-efficacy related to assisting someone at risk for suicide. In contrast, control participants made no significant improvement except in their self-perceived ability to tell if someone close to them is at risk for suicide. Results of our sensitivity analysis were similar for overall and all individual self-efficacy items. Nine (13 %) participants offered qualitative comments, which confirmed the perception of self-efficacy as a result of the intervention. Comments also frequently contained terms like “everyone” and “we”; this expanded on quantitative findings suggesting a sense of not just individual- but also community-level efficacy in addressing suicide prevention.

**Table 3:** Joint display linking quantitative results and qualitative comments related to self-efficacy

Variable	Group	Pre score	Post score	*P*-value	Comments about intervention
Self-efficacy (total)	Control	3.25 ± 0.89	3.54 ± 0.85	0.17	“Everyone can participate in improving the suicidal problem.” (E2_002) “Suicide CAN be prevented.” (E2_011) “Nobody else is going to take care of this ‘problem.’ We all need to help one another instead of being apathetic.” (E2_027)
	Intervention	3.30 ± 0.79	4.09 ± 0.77	<0.001	
Confident in helping someone with a mental health concern	Control	3.16 ± 1.05	3.47 ± 1.21	0.25	
	Intervention	3.30 ± 1.11	4.22 ± 0.81	<0.001	
Would be easy to directly ask question	Control	3.21 ± 1.42	3.44 ± 1.37	0.49	Mixed methods interpretation
	Intervention	3.26 ± 1.43	3.94 ± 1.12	<0.01	
Can persuade someone to get help	Control	3.53 ± 1.03	3.68 ± 0.77	0.49	Qualitative comments expand on quantitative results.
	Intervention	3.73 ± 0.91	4.22 ± 0.94	<0.01	
Can tell if someone close is at risk	Control	3.08 ± 1.04	3.56 ± 0.82	0.04	
	Intervention	2.95 ± 1.07	4.00 ± 0.82	<0.001	

Score range was on a five-point scale from 1 (“strongly disagree”) to 5 (“strongly agree”)

Table 4 contains mixed methods findings regarding social norms. Intervention participants showed significant increase in overall treatment-promoting social norms, and their individual beliefs that most people thinking about suicide would approve of—as well as actually seek—help from a mental health provider. No improvement was observed in control participants. Results of our sensitivity analysis were similar for overall and individual social norms except approval of seeking help, which became marginally significant (*p* = 0.06). Thirty-one (46 %) participants offered qualitative comments, which *expanded* on quantitative findings. These expansions included comments that culture and the political environment contribute to a sense of apathy and lack of concern about suicide as a health problem.

**Table 4:** Joint display linking quantitative results and qualitative comments related to social norms

Variable	Group	Pre score	Post score	*P*-value	Comments about intervention
Social norms (total)	Control	2.88 ± 0.86	3.26 ± 0.98	0.08	“Cultural background [is] contributing to the problem.” (E2_005) “Politics should be more concerned and involved.” (E2_013) “We need different perspectives (from other cultures) to improve our situation in Japan.” (E2_028) “I felt that it is important to further promote this film in Japan. I feel that it is necessary to spread knowledge that there are things that can’t be excused by saying ‘This is a Japanese national trait.” (J1_012)
	Intervention	2.69 ± 1.02	3.21 ± 1.03	0.01	
Most people would seek help from a mental health provider	Control	2.66 ± 0.85	3.06 ± 1.01	0.07	
	Intervention	2.38 ± 1.10	2.89 ± 1.18	0.01	
Most people would approve of seeking help from a mental health provider	Control	3.10 ± 1.06	3.47 ± 1.08	0.15	Mixed methods interpretation
	Intervention	3.00 ± 1.22	3.54 ± 1.20	0.01	Qualitative comments expand on quantitative results

Score range was on a five-point scale from 1 (“strongly disagree”) to 5 (“strongly agree”)

Table 5 presents results on the difference between intervention and control groups in change of scale scores. In adjusted models, the mean improvement in gatekeeper behavior (0.52 points; 95%CI = 0.28–0.75 points) and self-efficacy (0.55 points; 95%CI = 0.32–0.79 points) scores was significantly greater in the intervention group than control group. Change in social norms was not significantly different between intervention and control groups.

**Table 5:** Group differences in change in scale scores for gatekeeper behavior, self-efficacy, and social norms

Scale	Unadjusted	Adjusted^a^
	Mean difference (95 % CI) between intervention and control groups in change in scale scores	
Gatekeeper Behavior	0.52 (0.28,0.75)	0.52 (0.28,0.75)^b^
Self-Efficacy	0.50 (0.27,0.73)	0.55 (0.32,0.79)^c^
Social Norms	0.05 (-0.31,0.41	0.02 (-0.38,0.43)^d^

Table values indicate differences in score change between groups as calculated using regression models, with a positive value favoring the intervention group. All three scales had a 4-point range from a low of 1 to a high of 5

^a^Regression model utilized purposeful forward stepwise selection. Age, gender, race/ethnicity, education, whether a health care provider, number of close relations at risk for suicide, and whether seen/talked to a mental health provider in last 12 months were all examined as potential confounders; ^b^ Adjusted for baseline score; ^c^ Adjusted for baseline score and number of close relations; ^d^ Adjusted for baseline score, education, number of close relations, age, gender, race/ethnicity, health care provider, and seen a mental health provider

## Discussion

In this controlled intervention of 106 adults, we found that a brief, multi-modal intervention (film viewing, QPR skills training, and interactive discussion) does promote positive gatekeeper behaviors and self-efficacy for suicide prevention in at-risk ethnic minority population of Japanese Americans. Primary results showed significant improvements in all outcome measures in the intervention group: higher confidence in all three types of intended gatekeeper behavior, all four measures of self-efficacy, and both measures of social norms relevant to suicide prevention. In our sensitivity analysis, two outcomes became marginally significant while all other results remained the same. Change in these types of factors are meaningful and emphasized in theoretical models as important to creating health behavior change [33]. In fully adjusted statistical models, intervention group improvements were above-and-beyond the gains seen in control group for all a priori primary outcomes. Improvements were statistically significant even when participants showed high baseline levels of confidence, as was the case with gatekeeper behavior. The separate collection of qualitative data, and integration with the quantitative survey constructs confirmed and expanded understanding about the benefits of the intervention.

Although this was a non-randomized study for the very practical reason that the intervention was open to the public, participants in the intervention and control groups were highly similar other than they chose different events to attend. This was apparent for demographic characteristics we measured. But we also suspect it is the case for unmeasured health-promoting behavior that can confound observational studies [34] because both conditions received the same type of publicity and required similar levels of motivation for participation. From the perspective of the attendees, the control condition was itself an “intervention,” and some indication of the active component to it (eg, education on risk factors for suicide) might be indicated by our results showing increased self-efficacy a propos risk detection in the control group. Other key study strengths included a high survey response rate, confirmatory and expanding qualitative findings based on robust mixed methods procedures, and rigorous adaptation of study measures and materials into Japanese.

Three unique features of this study bear emphasis. First, the intervention was effective in a *high-risk ethnic minority population*. The few existing reports of suicide prevention interventions in at-risk minority adults that include a component of gatekeeper training have focused on indigenous populations, and results have been mixed [35, 36]. Second, the intervention targeted members of the *lay community.* Most prior studies of gatekeeper training have been conducted among individuals with some professional background and in their workplace setting, such as clinicians or school staff [37, 38]. Likewise, use of film for health education has traditionally targeted health care professional trainees [39–44]. Third, the intervention was *brief*. All told, it required just two hours, similar in length to standard QPR training. Other gatekeeper training programs such as Mental Health First Aid and ASIST require one to two full days of instruction, [15, 16, 26] which we see as too long to expect significant participation levels from the general public.

Several limitations of this study provide guidance for future work. First, selection bias is possible given the voluntary nature of the intervention. In an even more general community sample, it is possible that the effect size of our intervention is smaller. But it could also be even larger since our sample had high baseline levels of knowledge, experience, and confidence. Indeed, in a study of QPR gatekeeper training of staff at Department of Veterans Affairs (VA) hospitals for military veterans, results showed efficacy among clerical staff but not those with clinical backgrounds [19], which we believe may represent a ceiling effect among those with pre-existing expertise. Second, the intervention did not result in significantly more improvement than the control condition in social norms favoring help-seeking. It may be that changing such perceptions requires more repeated exposures or an altogether different intervention. Third, because we evaluated participants immediately after the intervention, the durability of effects is unknown. Fourth, evaluation of reduction in suicidal behavior and other objective behavioral indicators (eg, mental health treatment referral rates) remains for future study. Outcomes were based on self-report and blinding of research staff was not employed, which makes demand characteristics possible [45], although our active control likely reduced that risk. Finally, testing of this intervention in other settings and populations will require judicious selection of an appropriate film, an admittedly subjective choice for which we cannot provide empirical guidance. Appropriate film selection is not a trivial issue as studies have indicated that portrayal of suicide in film can also influence viewers’ subsequent behavior in negative ways, the most extreme of which is imitative suicidal behavior [46]. Thus, we believe subsequent work is needed to identify a list of criteria to guide film selection.

## Conclusions

This study provides strong mixed methods evidence of the preliminary efficacy of a brief, multimodal gatekeeper training in promoting positive gatekeeper behaviors and self-efficacy for suicide prevention in an at-risk ethnic minority population of Japanese Americans. We hope these promising findings provide stimulus for expanded evaluation of similar interventions using similarly rigorous mixed methods, as well as longitudinal and randomized study designs in other settings and populations.

## Abbreviations

ASIST, Applied Suicide Intervention Skills Training; CIVIC, Center to Improve Veteran Involvement in Care; HSR&D, Health Service Research & Development; QPR, Question, Persuade, and Refer; SAMHSA, Substance Abuse & Mental Health Service Administration; SOS, Signs of Suicide; U.S., United States; VA, Department of Veterans Affairs

## References

[1] Mathers CD, Loncar D (2006). Projections of global mortality and burden of disease from 2002 to 2030. PLoS Med.

[2] Center for Disease Control and Prevention. (n.d.-a). Connectedness as a Strategic Direction for the Prevention of Suicidal Behavior. 2015. Retrieved August 19, 2015, from https://www.cdc.gov/violenceprevention/pdf/suicide_strategic_direction_full_version-a.pdf

[3] Center for Disease Control and Prevention. (n.d.-b). Fatal Injury Reports | WISQARS | Injury Center | CDC. 2015. Retrieved August 19, 2015, from http://www.cdc.gov/injury/wisqars/fatal_injury_reports.html

[4] Chan CH, Caine ED, Chang SS, Lee WJ, Cha ES, Yip PSF (2015). The impact of improving suicide death classification in South Korea: a comparison with Japan and Hong Kong. PLoS One.

[5] Hoeffel et al. 2012. The Asian Population: 2010–2010 Census Briefs. Retrieved January 19, 2016, from http://www.census.gov/prod/cen2010/briefs/c2010br-11.pdf.

[6] Ahmedani BK, Stewart C, Simon GE, Lynch F, Lu CY, Waitzfelder BE, Williams K (2015). Racial/Ethnic differences in health care visits made before suicide attempt across the United States. Med Care.

[7] Vogel DL, Wade NG, Wester SR, Larson L, Hackler AH (2007). Seeking help from a mental health professional: the influence of one’s social network. J Clin Psychol.

[8] Sakamoto S, Tanaka E, Kameyama A, Takizawa T, Takizawa S, Fujishima S, Ono Y (2014). The effects of suicide prevention measures reported through a psychoeducational video: a practice in Japan. Int J Soc Psychiatry.

[9] Barton AL, Hirsch JK, Lovejoy MC (2013). Peer response to messages of distress. Crisis.

[10] Institute of Medicine. (n.d.). Reducing Suicide: A National Imperative. 2002. Retrieved from 2002. http://www.nap.edu/catalog/10398/reducing-suicide-a-national-imperative

[11] Bartlett H, Travers C, Cartwright C (2008). Evaluation of a project to raise community awareness of suicide risk among older men. J Ment Health.

[12] Cross WF, Seaburn D, Gibbs D, Schmeelk-Cone K, White AM, Caine ED (2011). Does practice make perfect? A randomized control trial of behavioral rehearsal on suicide prevention gatekeeper skills. J Prim Prev.

[13] Osteen PJ, Frey JJ, Ko J (2014). Advancing Training to Identify, Intervene, and Follow Up with Individuals at Risk for Suicide Through Research. Am J Prev Med.

[14] Aseltine RH, James A, Schilling EA, Glanovsky J (2007). Evaluating the SOS suicide prevention program: a replication and extension. BMC Public Health.

[15] Gould MS, Cross W, Pisani AR, Munfakh JL, Kleinman M (2013). Impact of Applied Suicide Intervention Skills Training on the National Suicide Prevention Lifeline. Suicide Life Threat Behav.

[16] Kitchener BA, Jorm AF (2002). Mental health first aid training for the public: evaluation of effects on knowledge, attitudes and helping behavior. BMC Psychiatry.

[17] Wyman PA, Brown CH, Inman J, Cross W, Schmeelk-Cone K, Guo J, Pena JB (2008). Randomized Trial of a Gatekeeper Program for Suicide Prevention: 1-year Impact on Secondary School Staff. J Consult Clin Psychol.

[18] Intervention Summary - QPR Gatekeeper Training for Suicide Prevention. (n.d.). 2012. Retrieved July 5, 2016, from http://legacy.nreppadmin.net/ViewIntervention.aspx?id=299

[19] Matthieu MM, Cross W, Batres AR, Flora CM, Knox KL (2008). Evaluation of gatekeeper training for suicide prevention in veterans. Arch Suicide Res.

[20] Cross W, Matthieu MM, Cerel J, Knox KL (2007). Proximate Outcomes of Gatekeeper Training for Suicide Prevention in the Workplace. Suicide Life Threat Behav.

[21] Cross W, Matthieu MM, Lezine D, Knox KL (2010). Does a brief suicide prevention gatekeeper training program enhance observed skills?. Crisis.

[22] Klimes-Dougan B, Yuan C, Lee S, Houri AK (2009). Suicide prevention with adolescents: considering potential benefits and untoward effects of public service announcements. Crisis.

[23] Sargent JD, Beach ML, Dalton MA, Ernstoff LT, Gibson JJ, Tickle JJ, Heatherton TF (2004). Effect of parental R-rated movie restriction on adolescent smoking initiation: a prospective study. Pediatrics.

[24] Teo AR, Stufflebam KW, Lu F, Fetters MD (2015). Use of a public film event to promote understanding and help seeking for social withdrawal. Asia-Pacific Psychiatry: Official Journal of the Pacific Rim College of Psychiatrists.

[25] Boomachine. (2013). SAVING 10,000 - Winning a War on Suicide in Japan - 自殺者1万人を救う戦い - Japanese Documentary. Retrieved from https://www.youtube.com/watch?v=oo0SHLxc2d0

[26] Intervention Summary - Mental Health First Aid. (n.d.) 2012. Retrieved July 5, 2016, from http://legacy.nreppadmin.net/ViewIntervention.aspx?id=321

[27] Lawrence MT, Ureda JR (1990). Student recognition of and response to suicidal peers. Suicide Life Threat Behav.

[28] Lapinski MK, Rimal RN (2005). An Explication of Social Norms. Commun Theory.

[29] Yun D, Silk KJ (2011). Social norms, self-identity, and attention to social comparison information in the context of exercise and healthy diet behavior. Health Commun.

[30] Fetters MD, Freshwater D (2015). The 1 + 1 = 3 Integration Challenge. J Mixed Methods Res.

[31] Guetterman TC, Fetters MD, Creswell JW (2015). Integrating Quantitative and Qualitative Results in Health Science Mixed Methods Research Through Joint Displays. Ann Fam Med.

[32] Fetters MD, Curry LA, Creswell JW (2013). Achieving Integration in Mixed Methods Designs—Principles and Practices. Health Serv Res.

[33] Riekert KA, Ockene JK, Pbert L (2013). The Handbook of Health Behavior Change.

[34] Shrank WH, Patrick AR, Brookhart MA (2011). Healthy user and related biases in observational studies of preventive interventions: a primer for physicians. J Gen Intern Med.

[35] Clifford AC, Doran CM, Tsey K (2013). A systematic review of suicide prevention interventions targeting indigenous peoples in Australia, United States, Canada and New Zealand. BMC Public Health.

[36] Sareen J, Isaak C, Bolton S-L, Enns MW, Elias B, Deane F, Katz LY (2013). Gatekeeper Training for Suicide Prevention in First Nations Community Members: A Randomized Controlled Trial. Depress Anxiety.

[37] Pisani AR, Cross WF, Watts A, Conner K (2012). Evaluation of the Commitment to Living (CTL) curriculum: a 3-hour training for mental health professionals to address suicide risk. Crisis.

[38] Walsh E, Hooven C, Kronick B (2013). School-Wide Staff and Faculty Training in Suicide Risk Awareness: Successes and Challenges. J Child Adolesc Psychiatr Nurs.

[39] Alexander M, Lenahan P, Pavlov A. Cinemeducation: A Comprehensive Guide to Using Film in Medical Education. Radcliffe Publishing; 2005

[40] Bird L, Marti J (2009). Red carpet reception for inaugural IEPA Short Film Festival. Early Interv Psychiatry.

[41] Kalra G (2012). Talking about stigma towards mental health professionals with psychiatry trainees: A movie club approach. Asian J Psychiatry.

[42] Kerby J, Calton T, DiMambro B, Flood C, Glazebrook C (2008). Anti-stigma films and medical students’ attitudes towards mental illness and psychiatry: Randomised controlled trial. Psychiatr Bull.

[43] Masters JC (2005). Hollywood in the classroom: using feature films to teach. Nurse Educ.

[44] Rosenstock J (2003). Beyond A Beautiful Mind: Film Choices for Teaching Schizophrenia. Acad Psychiatry.

[45] Nichols AL, Maner JK (2008). The good-subject effect: investigating participant demand characteristics. J Gen Psychol.

[46] Till B, Tran US, Voracek M, Sonneck G, Niederkrotenthaler T (2014). Associations between film preferences and risk factors for suicide: an online survey. PLoS One.

